# Mrs. Garrett Anderson and the Midwives

**Published:** 1898-05-07

**Authors:** 


### MRS. GARRETT ANDERSON AND THE MIDWIVES.

In a long letter to the *Times*, Mrs. Garrett Anderson, M.D., reviews the objections to the Midwives' Registration Bill, which is down for its second reading in the House of Commons on May 11th. Mrs. Garrett Anderson goes at once to the very centre of things when she says: "The Bill has two objects, one which is mentioned in the preamble, and one which is not," the latter being "improving and legalising the position of midwives." She points out with great force that so far from insuring a superior training for the midwives who actually attend the poor, "skilful and educated women . . . would raise their fees and give up the nursing, and the poorest women, for whose benefit the Bill is said to be planned, would lose the chance of securing their services." Mrs. Garrett Anderson goes on to say that the question of the position of midwives "lies at the core of the whole matter. It is the true basis on which the agitation for registration rests, on the part of the promoters, though, in the view of their social allies, the Bill is simply an attempt to ameliorate the condition of the poor." Here, then, we have laid bare by Mrs. Anderson the real spring and motive of this agitation, the promoters of which "are working for complete independence and for a position of professional dignity upon a three months' training, and for nothing less." Yet they and their "social allies" are trying to persuade Parliament that their Bill is simply an attempt to ameliorate the condition of the poor! "What the poor need," says Mrs. Anderson, "and what the midwives aim at getting are very different things. The poor want cheap attendance and nursing in simple midwifery, and to have the security of a thoroughly-trained practitioner at call for difficulties. The midwives aim at becoming practitioners of midwifery themselves, and at practising independently." That is the kernel of the matter. The door to the medical profession is as open to women as it is to men—if they will study for five years—and these ladies have no excuse whatever for asking Parliament to grant them this "professional dignity" on the strength of a three months' training. Their only reasonable plea for appealing to Parliament for registration is their statement that it is for the good of the poor. But Mrs. Garrett Anderson's letter shows this to be a mere excuse, and that their real aim is to improve their own "position."

